# Impact of Silanization Parameters and Antibody Immobilization Strategy on Binding Capacity of Photonic Ring Resonators

**DOI:** 10.3390/s20113163

**Published:** 2020-06-02

**Authors:** Nina Bjørk Arnfinnsdottir, Cole A. Chapman, Ryan C. Bailey, Astrid Aksnes, Bjørn Torger Stokke

**Affiliations:** 1 Department of Physics, Center for Quantum Spintronics, NTNU Norwegian University of Science and Technology, NO-7491 Trondheim, Norway; 2 Department of Chemistry, University of Michigan, 930 North University Avenue, Ann Arbor, MI 48109, USA; coleac@umich.edu (C.A.C.); ryancb@umich.edu (R.C.B.); 3 Department of Electronic Systems, NTNU Norwegian University of Science and Technology, Trondheim NO-7491, Norway; astrid.aksnes@ntnu.no; 4 Department of Physics, Division of Biophysics and Medical Technology, NTNU Norwegian University of Science and Technology, NO-7491 Trondheim, Norway; bjorn.stokke@ntnu.no

**Keywords:** characterization, APTES, biosensors, AFM, ring resonator

## Abstract

Ring resonator-based biosensors have found widespread application as the transducing principle in “lab-on-a-chip” platforms due to their sensitivity, small size and support for multiplexed sensing. Their sensitivity is, however, not inherently selective towards biomarkers, and surface functionalization of the sensors is key in transforming the sensitivity to be specific for a particular biomarker. There is currently no consensus on process parameters for optimized functionalization of these sensors. Moreover, the procedures are typically optimized on flat silicon oxide substrates as test systems prior to applying the procedure to the actual sensor. Here we present what is, to our knowledge, the first comparison of optimization of silanization on flat silicon oxide substrates to results of protein capture on sensors where all parameters of two conjugation protocols are tested on both platforms. The conjugation protocols differed in the chosen silanization solvents and protein immobilization strategy. The data show that selection of acetic acid as the solvent in the silanization step generally yields a higher protein binding capacity for C-reactive protein (CRP) onto anti-CRP functionalized ring resonator sensors than using ethanol as the solvent. Furthermore, using the BS3 linker resulted in more consistent protein binding capacity across the silanization parameters tested. Overall, the data indicate that selection of parameters in the silanization and immobilization protocols harbor potential for improved biosensor binding capacity and should therefore be included as an essential part of the biosensor development process.

## 1. Introduction

The basis for diagnosis and monitoring of progression of disease is through quantitative determination of biologically relevant material such as various macromolecules or signaling molecules, possible pathogens, e.g., viruses and bacteria, or cells. Traditionally, these molecular parameters have been quantified in centralized laboratories applying processes that depend both on relevant infrastructure as well as personnel. Recently, there has been an increasing interest in the development of biosensors that translate the presence of such analytes reliably into relevant parameters with shorter acquisition time, lower sample volume requirements and in a way that is possible to deploy in point-of-care settings [1]. Such fast and compact biosensors are often referred to as “lab-on-a-chip” technology. In addition to medical diagnostic applications, these toolboxes can be tailormade as important research tools for identification and evaluation of candidate drugs in the pharmaceutical industry [2,3], for fundamental research issues, or for monitoring of safety and environmental purposes [4,5,6]. Various transducing principles are applied in lab on a chip biosensors, such as electrochemical [7] (e.g., amperometric [8], conductometric [9]), based on optical principles [10,11] (photonic resonators, photonic crystals, fluorescence-based), plasmonic [12,13] (surface plasmon resonance, localized surface plasmons), mechanical [14], acoustic [15], nanopore [16,17,18], and others. Although highly sensitive, these transducing principles are not inherently selective towards any distinct biomarker. The use of highly selective recognition molecules such as antibodies, fragments of antibodies, affibodies, aptamers or others is therefore required to ensure that the measured sensor signal is a result of a highly specific binding event. Thus, immobilization of recognition molecules on the sensor surface is key in exploitation of the sensitivity of the transducing principles by ensuring selectivity in the signal generation. Biofunctionalization of a sensor is not a trivial task, and there are many aspects needing attention: the chemical functionalization strategies available for the actual sensor material, the type of capture molecule wanted, the time and cost of the functionalization, the robustness of the functionalization and so on. Stable and reproducible immobilization without functional damage of the recognition molecules typically requires covalent binding to the sensor. For such covalent immobilization, one must first overcome the inherent limitation in the lack of biologically reactive binding sites on inorganic sensor substrates. This is accomplished by functionalizing the sensor substrates with polymer films that introduces molecular groups to the surface that can subsequently be used as anchors for further functionalization. On gold-coated sensor surfaces, this is typically done with thiol-ended self-assembled monolayers (SAMs), while silanization is often the method of choice for glass- or silicon-based sensors [19]. These SAMs and silane films typically introduces amino-, thiol- carboxyl- or epoxy- groups to the substrate that can subsequently be used to covalently immobilize capture probes either through a direct covalent bond induced through carbodiimide chemistry [20] or via the use of a linker such as bis(sulfosuccinimidyl)suberate (BS3) [21,22] or glutaraldehyde [23,24].

Here, we report on direct comparisons of multiple controlled immobilization protocols for antibodies onto a sensor based on photonic ring resonators as the transducing principle. The selection of this readout platform is based on the increased interest in applying such photonic elements in biosensors, due to their small size and high sensitivity combined with possibilities for multiplexing and mass production using methods already established by the semiconductor industry. In a typical ring resonator-based sensor, light is coupled into a straight waveguide and a ring resonator. The light in the waveguide is transferred and resonates in the ring resonator if the resonance condition given by

*m*λ = 2πr*n_eff_* (1)

is fulfilled. Here, *m* is an integer, *λ* is the wavelength of light and *n_eff_* is the effective refractive index experienced by the light resonant in the ring. The actual value of *n_eff_* depends on the resonator materials and geometry, in addition to the conditions in the ring’s immediate surroundings as experienced by the evanescent field. As the ring resonator material and geometry remain constant during an experiment, photonic ring resonators are very sensitive to changes in refractive index near the ring surface, and small refractive index changes can be detected as a shift in the resonating wavelength. By the immobilization of recognition molecules on the sensor substrate, the subsequent binding of target molecules to the capture probes results in changes in the refractive index probed by the evanescent field. This changes the resonance conditions and gives a shift in the resonant wavelength that is both highly specific and target concentration dependent [25,26].

Silicon oxides are commonly exploited materials for fabrication of ring resonators [26]. The typical choice of surface functionalization of silicon oxide-based sensors is silanization, although other options such as zwitterionic polymers [27] are available. During silanization, reactive hydroxyl groups on the substrate surface reacts with methoxy or ethoxy residues on the silane molecule, forming a covalent bond. The other end of the silane molecule consists of a carbon linker chain and a reactive residue subsequently used for further functionalization. A plethora of organosilanes are commercially available, and the selection of silane typically depends on the length of the linker chain and the desired reactive residue for further functionalization. Although different linker lengths are available, these are mostly significantly shorter than the penetration depth of the evanescent field of the ring resonator. Amino-terminated silanes are widely employed due their versatility in supporting conjugation with various abundant moieties, e.g., -COOH. Due to its low cost and robustness, the introduction of amino groups through silanization with (3-Aminopropyl)triethoxysilane (APTES) is a common choice [20].

Silanization protocols can be described as consisting of four main steps: cleaning and activation of the substrate to maximize the number of reactive hydroxyl groups on the substrate, silanization performed in either vapor or liquid phase, and rinsing for the removal of unbound silanes, before a final (and sometimes optional) curing step. There are many attempts to optimize protocols reported in the literature. These tend to either compare similar silanes, e.g., amino-terminated silanes, or compare different silanization protocols for the same silane, e.g., APTES. In this context, it is worth mentioning that the details in each step, such as time, temperature, choice of silanization method, choice of solvent and silane concentration varies significantly in the literature. One example here is the reported improved hydrolytically stable films over a larger range of pH values achieved by replacing APTES with 11 aminoundecyltriethoxysilane (AUTES) [28]. Another example is the reported increased density of amino groups on the silane film achieved by replacing APTES with (3-aminopropyl)diethoxymethylsilane (APRDMS) [29], and associated identification of the silane molecular structure to control the hydrophobicity of the silane film [30]. These results were, however, achieved by quite dissimilar silanization protocols. Two of these results were obtained through vapor deposition of the silanes, one at room temperature for 4 hours at a pressure of 1.6 Torr [28], the other at 150 °C for five min at a pressure of 2–3 Torr [30]. The third was performed by liquid deposition of 1% silane in a anhydrous toluene [29]. The experimental details of these studies exemplify that optimized protocols can be achieved in different ways, and that comparing silanization results are therefore not necessarily straight forward. This is of interest, as what constitutes successful immobilization has not been properly defined. Although some traits, such as stable, durable and repeatable immobilization, retention of antibody activity, and avoidance of nonspecific binding, are often mentioned in the literature, the search for an optimized surface functionalization protocol for silanization of silicon oxide has not givena definitive answer.

Furthermore, optimization is also commonly performed on flat silicon oxide substrates, and not on a functioning sensor. Properties of the silane film itself are selected as the optimization criterion, although some groups also report on immobilization of proteins [31] or nanoparticles [28] onto these films as part of the optimization process. The most common techniques used for silane film characterization are ellipsometry [28,32,33,34,35,36] for film thickness measurements, atomic force microscopy (AFM) [28,31,32,33,34,35,36,37,38,39] for topological imaging and average surface roughness, water contact angle (WCA) [28,32,33,34,35,37] measurements for surface hydrophobicity, and X-ray photoelectron spectroscopy (XPS) [34,38,39] or Fourier-transform infrared spectroscopy (FTIR) [33,34,39] for chemical characterization of the silane film. In addition, spectroscopic methods for determining the surface density of amino groups or immobilized proteins have been reported [29,31]. Fluorescent signals from immobilized biomolecules can be used for characterization of the immobilization procedure as well [33,40].

The functionalization protocols used for functional ring resonator-based sensors reported in the literature also reflect variation in silanization protocols. Park et al. reported in 2013 on a label-free DNA aptamer sensor for the detection of human immunoglobin E [23] and human thrombin, with detection limits of 33 pM and 1.4 nM, respectively. Here, the ring resonator sensors are functionalized through liquid silanization with a 2% APTES solution in 95% ethanol for 2 h, with subsequent immobilization of aptamers using glutaraldehyde as a linker. Sabaté del Rio et al. report on DNA functionalization of ring resonators through copper-catalyzed chemistry on rings silanized with a 2% mixture of 11-aziduondecyltriethoxysilane in toluene and an overnight incubation [41]. These sensors where subsequently used for real-time and label-free monitoring of solid-phase recombinase polymerase amplification, with a limit of detection of 7.8 × 10^−13^ M. Both Valera et al. [21] and Graybill et al. [22] report on silanization with a 5% APTES solution in acetone for 5 min before using the BS3 linker to immobilize antibodies and aminated ssDNA capture probes respectively. Graybill reports on multiplexed detection of expressed microRNAs, while Valera reported on biosensing of monocyte chemotactic protein 1.

The Genalyte Maverick system is a commercialized and extensively characterized microring resonator system. The Bailey lab has consistently pioneered the development of this microring platform, particularly in understanding surface interactions, and using those interactions towards translational purposes. Initial work included characterization of the microring evanescent field through multilayer deposition [42], fine tuning parameters for the platform for functionalizing capture agents [43], and defining reproducible binding motifs for understanding association and dissociation events of functionalized surfaces [44]. Based on these early studies, this platform has been used to establish a variety of applications, including translational multiplexed assays [45], chromatographic detection systems [46], and small molecule analyses [47]. In both fundamental and applicative work, this platform has shown to be a highly sensitive system to elucidate the characteristics of biosensors needed for robust detection schemes.

The variations in optimized protocols based on experiments and characterization of silanization and protein immobilization on flat silicon oxide substrates and in protocols used on working ring resonator-based sensor platforms begs the question: is optimizing silanization on flat substrates worth the time and cost of performing these experiments in terms of optimized performance of the finished sensor? To our knowledge, there has been no previous report on any direct comparison between silanization characterization on wafer substrates and the binding capacity achieved on the finished ring resonator sensor set up. Here, we present the results of the characterization of aminosilanization with APTES of silicon wafer samples, followed by subsequent immobilization of anti-CRP antibodies, either by carbodiimide chemistry or the use of a BS3 linker. The different steps in the functionalization protocol are characterized by AFM imaging and XPS to obtain morphological and surface chemistry parameters. These different protocol parameters are also tested on the Genalyte Maverick system, and the binding capacity for CRP on these differently functionalized sensors are determined. The focus has been on elucidating correspondence between readout parameters from the wafer substrates characterization and the measured protein binding capacity of the sensor that can be used to streamline the optimization process for new chemistries on photonic ring resonator sensors.

## 2. Materials and Methods

The experiments were designed to compare the results of functionalized flat silicon oxide wafer pieces characterized using AFM with results from a multiplexed ring resonator sensor chip using an established sensor system (Maverick, Genalyte). The sensor chip was functionalized using the same functionalization parameters as the silicon wafer pieces. This was done in order to assess the usefulness of characterization of flat wafer “dummy” substrates as part of the process of optimizing the functionalization protocol of a biosensor. The functionalization protocols were therefore conducted as similarly as possible, but the chemical functionalization protocols for the ring resonator chips and the flat wafer substrates are described separately below due to the flow regime employed in the Genalyte set-up. The two main points of comparison are AFM roughness analysis for silanized wafer specimens compared to online monitoring of silanization on the Maverick platform and AFM roughness analysis of immobilized anti-CRP on wafer pieces compared to online monitoring of CRP capture on sensor chips functionalized with anti-CRP following the same parameters as the wafer pieces.

### 2.1. Chemical Functionalization and Characterization of Crystaline Silicon Substrates

Flat 0.8 × 0.8 cm square silicon test substrates were cut from a 4-inch crystalline silicon wafer using a scriber (DX-III, Dynatex, Santa Rosa, CA, USA).

#### 2.1.1. Silanization

The wafer pieces were plasma cleaned (Diener Electronics, 50% power, 50% O2-gas, 2 min) to remove contaminants, and subsequently silanized by immersion in a solution of (3-Aminopropyl)triethoxysilane (APTES, Sigma) diluted in either 96% ethanol (VWR) or 1 mM acetic acid (Sigma) in deionized water. The different combinations of APTES concentration and incubation times employed are shown in Table 1. After silanization, the samples were rinsed in deionized water before drying with nitrogen gas. The resulting silanized substrates were inspected using AFM imaging.

#### 2.1.2. Antibody Immobilization

To assess the effect of silanization parameters on antibody immobilization, anti-CRP antibodies were immobilized onto wafer pieces silanized using various parameters (Table 1). Antibody immobilization onto silanized substrates was achieved either by carbodiimine chemistry or by use of a bis(sulfosuccinimidyl)suberate (BS3) linker, giving a total of 12 combinations of parameters for antibody immobilization, all of which were inspected using AFM imaging.

For the carbodiimide chemistry, N-Hydroxysuccinimide (NHS, sigma Aldrich) was dissolved in phosphate buffered saline (PBS, Sigma) to a concentration of 3 mg/mL. N-(3-Dimethylaminopropyl)-N′-ethylcarbodiimide hydrochloride (EDC, sigma Aldrich) was diluted in MES-bufffer (Sigma, pH 5.5) to a concentration of 1.25 mg/mL. The CRP monoclonal antibody (Thermo Fisher) was diluted in MES-buffer to a final concentration of 0.1 mg/mL. Immediately before immobilization, the EDC and NHS solutions were added to the antibody solution and the final concentration of EDC and NHS was 12.5 µg/mL and 30 µg/mL, respectively. A 20 µL droplet of this EDC- and NHS-containing antibody solution was subsequently incubated on each wafer piece for 60 min. After incubation, the wafer pieces were rinsed in PBS and water before drying with N_2_ gas.

For the BS3 linker (Thermo Fisher), 20 µL of 2.85 mg/mL freshly dissolved BS3 linker in 2 mM acetic acid was deposited on the substrate and incubated for 3 min after silanization. Subsequently, a droplet of 0.1 mg/mL CRP monoclonal antibody in PBS was incubated on the BS3-activated substrate for 60 min before rinsing in PBS and deionized water and drying with N_2_ gas.

#### 2.1.3. AFM

Substrates were imaged in AC mode (tapping mode) in air on a Cypher AFM (Asylum Research) using a PPP-NCH-W tip (Nanosensors). Surface topographs in tapping mode were captured using a drive frequency of 0.98xresonance, amplitude of 1 V for topographs up to 5 × 5 micrometers with 512 × 512 data points. Surface roughness parameters, R_a_, of the resulting topographs were obtained using AR15 software package. For silanized substrates, the surface roughness parameters presented for each silanization parameter are the average of four samples, each imaged at four different locations. For substrates with immobilized antibodies, the surface roughness parameters are the average of four imaged areas on one sample substrate for each silanization condition.

#### 2.1.4. XPS

X-ray photoemission measurements were carried out using Mg Kα (hν = 1253.6 eV) X-ray source. Photoelectrons were collected at normal emission using a PHOIBOS 150 energy analyzer. XPS measurements were performed on three flat test samples, that were silanized with APTES using 1 mM acetic acid as the solvent and the silanization parameters presented in Table 1.

### 2.2. Chemical Functionalization of Sensor Chips and Online Measurements

#### 2.2.1. Online Silanization

Ring resonator waveguide chips were acquired from Genalyte. The Genalyte Maverick M1 system has been described extensively in the previous literature [21,43]. Briefly, we use an automated flow system across a single resonator chip cartridge system, consisting of a mylar gasket for fluid retention and a Teflon top where tubing can be implemented. Reagents flow across the chip at a defined flow rate and have also been discussed in previous literature [21]. Determination of silanization efficiency onto the ring resonator chips was performed through online flow experiments, and the sensor chips were not functionalized prior to these experiments. All steps used consistent flow rates of 30 µL/min. The assay steps were: (1) an initial rinse in silanization solvent for 5 min, (2) ranges of APTES content in silanization solution for 20 min, (3) a final rinse in silanization solvent. Silanization solutions were either 96% ethanol or 1 mM acetic acid, and the APTES content was either 1%, 2% or 4%. The results are reported as net average shifts, by subtracting the ring resonator shift in the pre-silanization condition from the signal after post-silanization rinse step.

#### 2.2.2. Functional Primary Binding Assays

To determine the effect of silanization parameters and immobilization chemistry, chips were functionalized with silane before anti-CRP was bound to the surface offline using either EDC/NHC-chemistry or BS3 linkers, as described previously [48]. Briefly, chips were silanized using either 96% ethanol or 1 mM acetic acid as the solvent, with 1%–4% APTES for 10 to 60 min (parameters as listed in Table 1). When using EDC as the linker, chips were spotted with a solution containing anti-CRP prepared at 0.25 mg/mL in 1xPBS solution and 5% glycerol. EDC and NHS were added to the spotting solution immediately before spotting at the same concentrations as described for the flat wafer substrates. For the BS3 linker, silanized substrates were incubated with BS3 (2.85 mg/mL in 2 mM acetic acid, 3 min) before being spotted with aqueous anti-CRP (0.25 mg/mL, 1xPBS, with 5% glycerol). All chips were incubated with anti-CRP for an hour before blocking with StartingBlock solution (Thermo Fisher).

To investigate how the different silanization parameters in combination with the two different antibody immobilization protocols affected the ring resonator sensor sensitivity to CRP capture, primary binding assays for CRP were conducted on the functionalized chips at a constant flow rate of 30 µL/min. The assay steps were: (1) 1× PBS, 0.5% BSA rinse for 5 min, (2) 2.5 µg/mL CRP in PBS-BSA for 10 min, (3) PBS-BSA rinse for 5 min. Comparison of final shifts were extracted in a similar way to the online silanization shifts.

## 3. Results and Discussion

### 3.1. Silanization of Flat Silicon Test Surfaces

**Figure 1:**
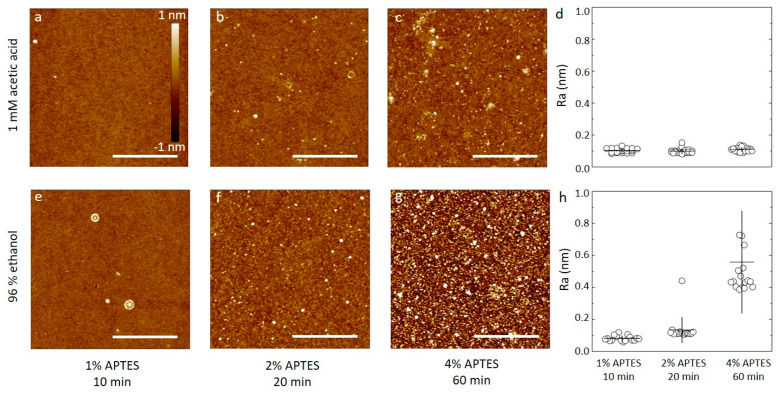
(**a**–**c**,**e**–**f**) A set of six AFM topographs of silicon oxide substrates silanized with APTES. The substrates are silanized using either 96% ethanol or 1 mM acetic acid as the solvent and the APTES concentration is varied from 1% to 4%, as indicated in the figure. The height scalebar for all topographs is shown in Figure 1a. Lateral scalebars are 1 µm. (**d**,**h**) The average surface roughness of silanized substrates prepared similar to the topographs in (**a**–**c**) and (**e**–**f**). The average is calculated based on four separate areas imaged on each of four samples prepared for each combination of solvent and APTES concentration parameters.

Silicon wafers were silanized by APTES at concentrations from 1% to 4% dissolved in aqueous 1 mM acetic acid or 96% ethanol for durations as summarized in Table 1. Selected AFM topographs of the obtained silanized silicon wafer substrates are shown in Figure 1 alongside the obtained surface roughness of the silanized surfaces. The AFM topographs of the silanized silicon wafers using dilute aqueous acetic acid as the solvent for the APTES show surfaces with only small height variations. These topographs show a small fraction of the surface containing nanosized domains with height about 1 nm from the mean. These domains are suggested to originate as a result of the silanization process, as APTES is known to both self-polymerize and to bond to oxidized substrates in several different ways, including multilayer formation (Figure S1, adapted from [20]). For the 1% APTES, 10 min silanization, there are very few nanosized domains in the 2.5 µm × 2.5 µm area of the topograph (Figure 1a). Increasing the APTES concentration to the 2% and duration of incubation to 20 min, increases the abundance of these domains to the order of 6–8 µm^−2^ (Figure 1b), and to 20–40 µm^−2^ for silanization at 4% for 60 min (Figure 1c). The roughness parameters estimated from the AFM topographs of the silicon wafers silanized using aqueous 1 mM acetic acid were observed to (0.10 ± 0.01) nm, (0.10 ± 0.01) nm and (0.11 ± 0.01) nm for the C_APTES_ = 1%, 10 min, C_APTES_ = 2%, 20 min, and C_APTES_ = 4%, 60 min, respectively (Figure 1d).

**Figure 2:**
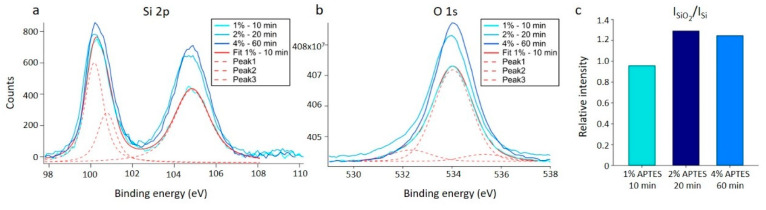
XP spectra results for crystalline silicon silanized with APTES using acetic acid as the silanization solvent. (**a**) Si 2p spectra. (**b**) O 1s spectra. (**a**,**b**) The results are displayed with the curve fitting results for the 1% APTES, 10 min silanization sample, which consisted of three separate peaks that combines to the overall fitted curve (Fit 1%—10 min). Similar curve fits were performed for the two additional silanization conditions, but the fitted curves to the data are not included in the graph. (**c**) Bar plot showing the relative intensities of the SiO2 peak at ~104.8 eV to the bulk silicon peak at ~100.5 eV. Results are calculated based on the fitted curves.

These estimates are not significantly different from (0.09 ± 0.01) nm observed for the plasma cleaned silicon wafer (Figure S2). To ensure that the samples were successfully silanized using this procedure, application of XPS on similarly functionalized samples was performed. The results provided clear evidence of silane film on the surface. Three silanized samples were analyzed using XPS, one for each of the silanization parameters for the solvent acetic acid presented in Table 1. There is an increase in intensity in the SiO_2_ peak at ~104.8 eV with increased silanization. In the oxygen signal, the same increase in intensity can be seen. This increased oxygen and silicon signal reflect an increase in silane on the substrates with increasing silane concentration and incubation time (Figure 2a,b). The relative ratio between the bulk Si peak at ~100.5 eV and the SiO_2_ peak at ~104.8 eV (Figure 2c) also shows an increase in the relative amount of Si-O bonds, indicating a larger amount of silane on the substrate. This is in agreement with reports on XPS measurements of silanization of crystalline silicon in the literature [49].

Thus, the silanization process using dilute acetic acid as the solvent yields overall a layer with roughness of 0.1 nm that is constant when increasing the concentration and duration of the silanization step. Additionally, in view of the observed tendency of some nanodomains, this indicate that the acetic acid solvent does not promote extensive APTES self-polymerization, and that a uniform layer can be formed using a certain range of silanization parameters. Although some nanodomains are observed in the AFM height topographs, it should also be noted that the height of these are in the order of a nm, e.g., within the same order of magnitude as the size of an APTES molecule, indicating that the nanodomains are not extensive self-polymerized APTES structures.

The appearance of the silanized silicon wafers using APTES in 96% ethanol differ from that using acetic acid solvent by showing increasing roughness with increasing incubation time and APTES concentration, whereas using a 1 mM solution of acetic acid results in substrates where the average surface roughness is relatively constant across the parameters investigated. The AFM topographs of the silanized silicon wafers using APTES in 96% ethanol shows an increasing fraction of the surface containing nanosized domains that also increases in size, with increasing C_APTES_ and incubation time. For the 1% APTES, 10 min silanization, there are very few nanosized domains in the 2.5 µm × 2.5 µm area of the topograph (Figure 1e), e.g., like that observed using acetic acid as the solvent. Increasing the APTES concentration to 2% and duration of incubation to 20 min, increases the abundance of these domains to the order of 30–50 µm^−2^ (Figure 1f), and to >100 µm^−2^ for silanization at 4% for 60 min (Figure 1g). The roughness parameters estimated from the AFM topographs of the silicon wafers silanized using 96% ethanol were observed to (0.08 ± 0.02) nm, (0.13 ± 0.08) nm and (0.55 ± 0.31) nm for the (C_APTES_ = 1%, t = 10 min), (C_APTES_ = 2%, t = 20 min), and (C_APTES_ = 4%, t = 60 min), respectively (Figure 1h). These estimates show that the combined lowest APTES concentration and duration of the incubation step yields a surface with roughness equal to the plasma cleaned wafer. Increasing C_APTES_ and duration of the incubation step yields surfaces with increasing roughness. Increasing extent of APTES self-polymerization is a possible mechanism leading to these structures.

### 3.2. Online Silanization of Ring Resonator Chips

**Figure 3:**
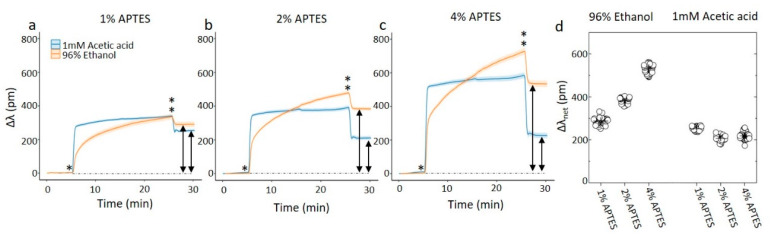
(**a**–**c**) On-chip monitoring of silanization of a ring resonator chip. The rings are exposed to the solvent for 5 min, before a solution of APTES and the solvent is flowed over the sensor for 20 min. The flow is returned to the solvent for 5 min before ending the experiment. The * denotes the time of APTES introduction to the channel while ** is the time point of return to the solvent without APTES. The graphs each represents the average of at least 47 ring resonators. (**d**) The average net shift, Δλ_net_ (arrows in the figure) of the resonant wavelength as a result of the silanization. The net shift is calculated as the difference between resonating wavelength before and after the silanization step.

The silanization processes using the two different solvents for the APTES were also performed on ring resonator sensor chips on the Genalyte system (Figure 3). In comparing the data, one should be aware that the silanization process as monitored by the ring resonator sensor chip differs from the process used to obtain the data above since the silanization solution is flowing over the chips at constant flow rate while the signal is recorded. The procedure consisted of a solvent rinse, followed by exposing the ring resonators to the silanization solution for 20 min, followed by a second solvent rinse, all at the same volumetric flow rates. The shift in the resonance data show different signatures when using 1mM acetic acid as the solvent as compared to 96% ethanol. For the 1 mM acetic acid solvent, the change in resonance frequency is initially quick, followed by a less rapid increase. The change in resonant wavelength, Δλ, from the flushing of the solution to the solvent in the second rinse is almost instantaneous, and its magnitude is reflecting the impact on the contribution to the refractive index in the solvent from the APTES on Δλ. The net resonance shift Δλ_net_ in the final rinse compared to the initial rinse, is (252 ± 8) pm for the 1% APTES, 10 min treatment, changing to (206 ± 11) pm and (218 ± 15) pm for the increasing C_APTES_ and durations, e.g., in the order of 210–250 pm without any clear overall trend for all the three C_APTES_. The Δλ shift from the solution to the final rinse is proportional to the C_APTES_.

The 96% ethanol solvent differs from that observed for 1 mM acetic acid solvent by showing profiles for the changes in Δλ with smaller initial change at the same APTES concentration, followed by a more strongly time-dependent increase. Like the case for silanization using acetic acid as the solvent, the change Δλ from the APTES and 96% ethanol solution to the final rinse is observed to be proportional to C_APTES_. The net resonance shift Δλ_net_ in the final rinse compared to the initial rinse, increases from (287 ± 16) pm for the 1% APTES, to (382 ± 11) pm for the 2% and further to (531 ± 20) pm for the 4% APTES. However, since the silanization appear to continue to develop during the whole 20 min duration of the APTES solution flushing, these values are reflecting a snapshot in the developing silanized surface. Self-polymerization of the APTES silane proceeding quite readily in ethanol is a possible reason for this.

The results presented above, including the ring resonator readout, display consistent trends reported in the literature related to C_APTES_ and incubation times. These results relate to differences in optimized silanization protocol parameters, even when screening a rather narrow set of parameters, i.e., liquid silanization with APTES with toluene as the solvent. It has been reported that a 2% APTES solution with immersion for 30 min at room temperature under nitrogen atmosphere gives a (local) maximum density of amino groups and subsequently the highest number of immobilized proteins on these substrates [31]. Similarly, a different group reports that when using the same parameters, the immersion time should be 1 hour for optimal protein immobilization [33]. Stable silane films with maximum protein adsorption have also been reported on substrates silanized with 50 mM APTES (ca. 1.2%) with a 12-hour immersion time at 90 °C [37]. When looking for the maximum density of amino groups and minimized silane film roughness (on the order of the underlying substrate), Howarter et al. reports that when testing 1%, 10% and 33% APTES solution, all films are essentially smooth and thin after 1 h [36]. They also observed that for the 1% APTES solution, increasing the silanization time above 1 h resulted in less smooth films. Another interesting observation is that the substrate cleaning and activation protocols can affect the final silane film [34]. This article also reports that rinsing the silanized substrates in 1 mM acetic acid results in thin uniform thin films across a range of parameters, even if the films do not appear thin and smooth before the acetic acid rinse [34].

### 3.3. Characterization of Antibody Immobilization on Wafer Substrates

**Figure 4:**
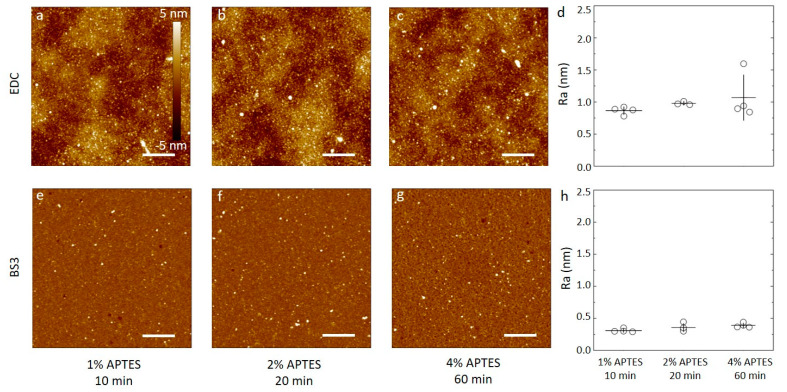
(**a**–**c**,**e**–**f**) AFM height topographs of anti-CRP antibodies immobilized on silicon substrates silanized using APTES dissolved in 1 mM acetic acid. (**a**–**c**) Height topographs of anti-CRP immobilized by carbodiimide chemistry (EDC) to substrates silanized using APTES concentrations and durations of (**a**) 1% APTES, 10 min, (**b**) 2% APTES, 20 min, and (**c**) 4% APTES, 60 min. (**d**) The average surface roughness obtained from 5 µm × 5 µm AFM topographs (n = 4, for each condition) of anti-CRP immobilized on silanized silicon. (**e**–**f**) Selected AFM height topographs of anti-CRP immobilized using the BS3 linker to silicon substrates silanized using APTES concentrations and durations of (**e**) 1% APTES, 10 min, (**f**) 2% APTES, 20 min, and (**g**) 4% APTES, 60 min. (**h**) The average surface roughness obtained from 5µm × 5 µm AFM topographs (n = 4, for each condition) of anti-CRP immobilized using BS3 on silanized silicon substrates). The height scalebar for all topographs are shown in Figure 4a. The lateral scalebars on the AFM topographs are 1 µm.

**Figure 5:**
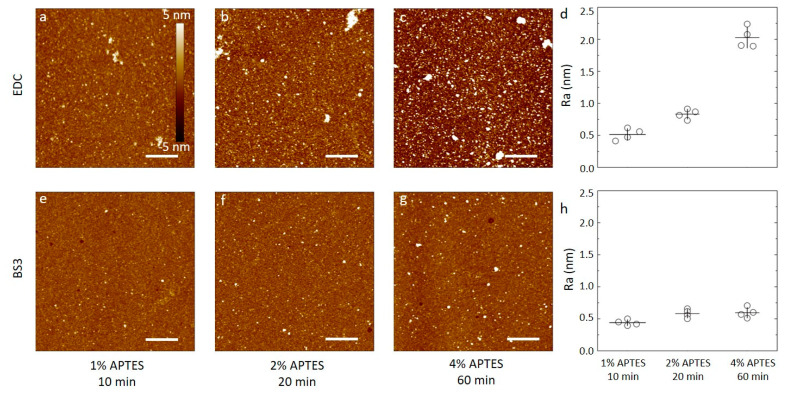
(**a**–**c**,**e**–**f**) AFM height topographs of anti-CRP antibodies immobilized on silicon substrates silanized using APTES dissolved in 96% ethanol. (**a**–**c**) Height topographs of anti-CRP immobilized by carbodiimide chemistry (EDC) to substrates silanized using APTES concentrations and durations of (**a**) 1% APTES, 10 min, (**b**) 2% APTES, 20 min, and (**c**) 4% APTES, 60 min. (**d**) The average surface roughness obtained from 5 µm × 5 µm AFM topographs (n = 4, for each condition) of anti-CRP immobilized on silanized silicon. (**e**–**f**) Selected AFM height topographs of anti-CRP immobilized using the BS3 linker to silicon substrates silanized using APTES concentrations and durations of (**e**) 1% APTES, 10 min, (**f**) 2% APTES, 20 min, and (**g**) 4% APTES, 60 min. (**h**) The average surface roughness obtained from 5 µm × 5 µm AFM topographs (n = 4, for each condition) of anti-CRP immobilized using BS3 on silanized silicon substrates. The height scalebar for all topographs are shown in Figure 4a. The lateral scalebars on the AFM topographs are 1 µm.

Samples of silicon wafers silanized by APTES using either 1 mM acetic acid or 96% ethanol under the various conditions described above were functionalized with anti-CRP antibody using either carbodiimide chemistry or BS3 linker, and the resulting surfaces were characterized by AFM. The overall impression from the AFM topographs (Figure 4 and Figure 5) are that the surface roughness is dependent on both silanization solvent used and the subsequent choice of linker to immobilize the antibodies.

For the APTES silanized surfaces using acetic acid solvent and the three silanization conditions, the data reveal the following features after the subsequent anti-CRP immobilization (Figure 4). In the case of using water soluble carbodiimide chemistry in the coupling reaction (Figure 4a–c), the AFM topographs reveal a small fraction of the area covered by small domains with a height in the order of 2–3 nm from the adjacent areas. Neither the fraction nor the size of these domains appear to be correlated with the increasing C_APTES_ or duration of the silanization step in the process step prior to the conjugation. In addition, there appears to be a longer spatial wavelength variation in the background of the AFM topographs. The obtained average surface roughness of the anti-CRP surfaces shows a small increase from (0.86 ± 0.06) nm to (0.98 ± 0.03) and further to (1.1 ± 0.3) nm for the silanization parameters (C_APTES_ = 1%, t = 10 min), (C_APTES_ = 2%, t = 20 min) and (C_APTES_ = 4%, t = 60 min), respectively (Figure 4d). The AFM topographs of the anti-CRP immobilized using the BS3 linker in the conjugation step (Figure 4e–g) reveal, similar to the EDC supported immobilization, a small fraction of the surface displaying nanosized domains with heights in the range 2–4 nm. The heights of these domains are larger than observed for the silanized surfaces, and thus emerge in the immobilization step. These heights are also comparable to the smallest dimension of an antibody, 14.5 nm × 8.5 nm × 4 nm [50], indicating that extensive aggregation of the antibodies are not induced in the immobilization process. The obtained average surface roughness of the anti-CRP surfaces shows a small increase from (0.31 ± 0.03) nm to (0.35 ± 0.06) nm and further to (0.39 ± 0.04) nm for the silanization parameters (C_APTES_ = 1%, t = 10 min), (C_APTES_ = 2%, t = 20 min) and C_APTES_ = 4%, t = 60 min), respectively (Figure 4h). These are all less than for the immobilization using EDC.

Similar analyses of the anti-CRP immobilized to the APTES silanized surfaces using ethanol as the solvent yield AFM topographs with distinguishable nanodomains with heights up to 5 nm that increases with the increasing C_APTES_, and silanization duration for both the EDC and BS3-based coupling strategies (Figure 5). The occurrence of these domains is observed to be more pronounced and increases more strongly with increasing concentration and silanization reaction time in the case of EDC mediated coupling than for the BS3 (Figure 5d,h). The obtained average surface roughness of the anti-CRP surfaces shows the largest increase in case of the EDC coupling to the silanized surfaces using 96% ethanol as the solvent, starting from (0.51 ± 0.09) nm to (0.83 ± 0.09) nm and further to (2.0 ± 0.2) nm for the silanization parameters (C_APTES_ = 1%, t = 10 min), (C_APTES_ = 2%, t = 20 min) and (C_APTES_ = 4%, t = 60 min), respectively (Figure 5d). When using BS3 coupling, the obtained average surface roughness of the anti-CRP functionalized surfaces shows a small increase from (0.44 ± 0.05) nm to (0.58 ± 0.07) nm and further to (0.60 ± 0.08) nm for the silanization parameters being (C_APTES_ = 1%, t = 10 min), (C_APTES_ = 2%, t = 20 min) and (C_APTES_ = 4%, t = 60 min), respectively (Figure 5h), which are all less than for the immobilization using EDC.

The surface roughness of the surfaces following immobilization with anti-CRP are all larger than for the underlying silanized silicon wafer, except for the silanization using 96% ethanol as the solvent with C_APTES_ = 4%, t = 60 min used as substrate for immobilizing anti-CRP employing BS3 as the linker. Although this may indicate that the surface roughness of the anti-CRP is largely determined in the immobilization process, the differences in roughness appear also to be related to the initial silanized layer. Using the BS3 linker, the surfaces of the immobilized antibodies to the silanized silicon substrates all have roughness in the range 0.34 to 0.6 nm, and only modestly increasing with the increased C_APTES_, and silanization time. The trend of changes in roughness with increased silanization time and concentration for silanized substrates appear thus to be mirrored in the roughness of the subsequently immobilized antibodies for all combinations except for the combination of ethanol as the silanization solvent and BS3 as the immobilization linker (Figure 5g).

### 3.4. On-Chip Capture of CRP

**Figure 6:**
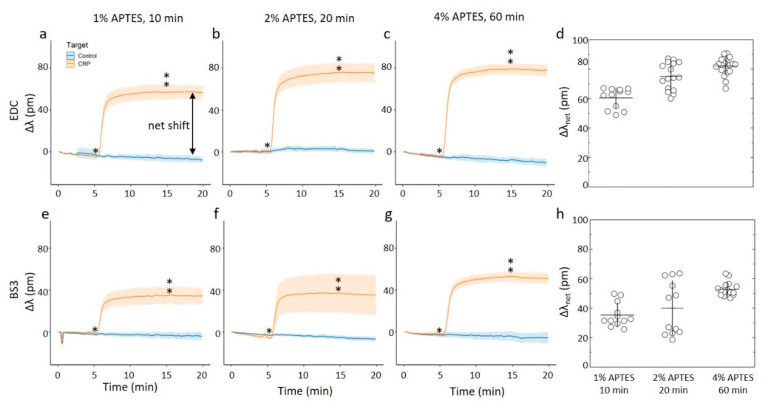
(**a**–**c**,**e**–**f**) Online measurements of CRP capture on ring resonator sensor chips silanized with APTES using 1 mM acetic acid as the silanization solvent. The assay steps consisted of a PBS-BSA rinse for 5 min before a 2.5 ug/mL CRP in BSA-PBS for 10 min and a final PBS-BSA rinse for 5 min. Single- and double-stars marks start and end of CRP in BSA-PBS step respectively. For these experiments, at least 12 rings were used for technical replicates. (**a**–**c**) CRP capture onto chips where the anti-CRP capture molecule has been immobilized using by carbodiimide chemistry onto chips silanized using APTES concentrations and durations of (**a**) 1% APTES, 10 min, (**b**) 2% APTES, 20 min, and (**c**) 4% APTES, 60 min. (**d**) The average net shift in resonance wavelength due to CRP capture onto the ring resonator chips with anti-CRP immobilized with by carbodiimide chemistry. (**e**–**g**) CRP capture onto chips where the anti-CRP capture molecule has been immobilized by the BS3 linker onto chips silanized using APTES concentrations and durations of (**e**) 1% APTES, 10 min, (**f**) 2% APTES, 20 min, and (**g**) 4% APTES, 60 min. (**h**) The average net shift in resonating wavelength due to CRP capture onto the ring resonator chips with anti-CRP immobilized with the BS3 linker.

**Figure 7:**
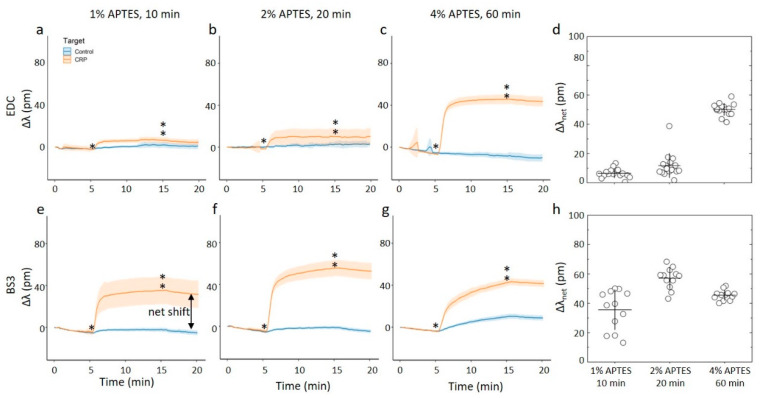
(**a**–**c**,**e**–**f**) Online measurements of CRP capture on ring resonator sensor chips silanized with APTES using 96% ethanol as the silanization solvent. The assay steps consisted of a PBS-BSA rinse for 5 min before a 2.5 ug/mL CRP in BSA-PBS for 10 min and a final PBS-BSA rinse for 5 min. Single- and double-stars mark the start and end of CRP in BSA-PBS step, respectively. For these experiments, at least 12 rings were used for technical replicates. (**a**–**c**) CRP capture onto chips where the anti-CRP capture molecule has been immobilized by carbodiimide chemistry onto chips silanized using APTES concentrations and durations of (**a**) 1% APTES, 10 min, (**b**) 2% APTES, 20 min, and (**c**) 4% APTES, 60 min. (**d**) The average net shift in resonance wavelength due to CRP capture onto the ring resonator chips with anti-CRP immobilized by carbodiimide chemistry. (**e**–**g**) CRP capture onto chips where the anti-CRP capture molecule has been immobilized by using the BS3 linker onto chips silanized using APTES concentrations and durations of (**e**) 1% APTES, 10 min, (**f**) 2% APTES, 20 min, and (**g**) 4% APTES, 60 min. (**h**) The average net shift in resonating wavelength due to CRP capture onto the ring resonator chips with anti-CRP immobilized with the BS3 linker.

For the on-chip measurements, both solvents and all silanization parameters where tested and combined with the two antibody immobilization strategies. The changes in the resonance frequency of these differently prepared chips were monitored when exposed to a continuously flowing solution, with the baseline being established for aqueous PBS-BSA buffer for 5 min, followed by aqueous 2.5 µg/mL CRP in PBS-BSA for 10 min, followed by a PBS-BSA buffer solution again (rinse). The relative shifts were determined throughout the experiments (Figure 6 and Figure 7). In contrast with the similar strategy employed for the silanization process (Figure 3), the rinsing step did not induce a reduction in the relative shift. This indicates that binding of CRP monitored in the presence of excess BSA is stable, and do not represent non-specific binding.

The results show the relative shift increasing from approximately 60 pm to 80 pm for the three combinations of silanization parameters tested for acetic acid as the silanization solvent and EDC as the coupling agent (Figure 6a–d). Within this particular immobilization protocol (e.g., using acetic acid as the solvent in the silanization step, and coupling of the anti-CRP employing EDC), there appears to be a correlation between the surface roughness of the surface with anti-CRP and the resonance shift when CRP is binding. This correlation can arise from an increased capacity to bind CRP per unit area the rougher the surface, the latter also indicating a larger anti-CRP effective density. The results of relative net shifts due to CRP-capture are the largest when compared to the set of investigated parameters in the immobilization protocol here. Changing the coupling agent to BS3 causes a reduction of the relative wavelength shift to approximately 40–60 pm, but the measured relative shift keeps comparable across the silanization parameters. This appears to fit well with the results of the silanization parameters, as the surface roughness of both the silanized substrates and antibodies immobilized on these substrates also are consistent across the silanization parameters.

For the chips silanized using ethanol as a solvent and EDC as the coupling agent (Figure 7a–d), the 10 min and 20 min duration of the silanization and subsequent immobilization of anti-CRP yielded chips that display far less shift when exposed to CRP. Changing the duration to 60 min and 4% APTES and subsequent EDC catalyzed immobilization, yielded chips displaying a net shift of about 50 pm when exposed due to CRP. This is about 60% of the binding capacity of the chip prepared using acetic acid as the solvent and identical silane concentration and duration. In other words, when using ethanol as the silanization solvent in combination with EDC, it is only the roughest surfaces that results in a substantial protein capture on chip. For the BS3 coupling agent used for the silanized surfaces using ethanol as the solvent, we observe an apparent non-monotonic change in the relative wavelength shift with increasing duration and concentration of the silane (Figure 7e–h). For the C_APTES_ = 1% applied for 10 min, the parameter Δλ_net_ for the CRP capture was observed to 37 pm. The ring-resonator readout increased to Δλ_net_ = 60 pm for C_APTES_ = 2% applied for 20 min, which was subsequently reduced to Δλ_net_ = 48 pm for C_APTES_ = 4% applied for 60 min. Although there is some experimental uncertainty, the overall trend of this non-monotonic readout appears significant.

## 4. Conclusions

The choice of silanization parameters (solvent, concentration and incubation time) and linker chemistry for antibody immobilization has been shown to impact both the surface roughness of silane and immobilized CPR antibodies on flat silicon test substrates, as well as affecting the binding capacity of CRP on a ring resonator-based setup. The impact of the choice of linker was larger in terms of absolute values on both the average surface roughness of immobilized antibodies on flat substrates and on the magnitude of the relative wavelength shift due to CRP capture than the choice of silanization parameters. Employing EDC coupling chemistry yielded the largest binding capacity for the CRP capture in terms of absolute value of net shift in the ring resonator. Please note that this coupling chemistry also resulted in the smallest binding capacity measured in this study. Meanwhile, BS3 gave CRP capture values that were much less influenced by the silanization parameters tested, indicating a higher potential for reproducible assay results. This consistency across the silanization parameters tested also indicates that the BS3 linker is more robust and is more likely than the EDC chemistry to produce robust arrays.

Some mirroring trends could be observed between roughness parameters of the silanized substrates, the roughness of immobilized antibodies and the CRP capture onto similarly functionalized ring resonator sensor chips. These trends indicate that in general, an increase in silane surface roughness due to increased C_APTES_ and incubation time gives rise to an increase in surface roughness in immobilized antibodies that also, in some instances, can be observed as an increase in captured CRP on a ring resonator-based sensor. These trends were more prominent when using acetic acid as the silanization solvent than ethanol. Although these trends are mirrored across different experiments, they do not give rise to an absolute value of either silane surface roughness or anti-CRP surface roughness being a boundary condition for significant CRP capture on a similarly functionalized ring resonator sensor.

Even though the AFM measurements on flat test substrates have been shown to be a reasonable platform to indicate which combination of parameters gives effective CRP capture on a ring resonator sensor, it is not a time-efficient strategy. The time spent for capturing AFM topographs of the flat test substrates with anti-CRP is comparable to the time spent immobilizing anti-CRP on ring resonator chips and subsequently monitoring the CRP capture on these chips. However, where the AFM results only indicate what might be an optimal immobilization strategy, the CRP capture experiments give an exact readout on which combination of parameters gives the highest sensor sensitivity. It is, therefore, our recommendation that for established sensor set ups, any optimization or change in surface chemistry should be tested directly on the sensor set up. If, however, the optimization is a part of the establishment of a new sensing platform where direct sensing is not yet possible, the use of AFM-based strategies offer some possibility of narrowing down which functionalization parameters that could be used on the sensor once the setup is complete. It is still vital to test this narrow set of parameters on the finished sensor, as capture of the desired biomarker onto the sensor is the one parameter that will distinguish a successful sensor from a failed experiment.

## Figures and Tables

**Table 1:** Overview of silanization parameters used in this study.

Solvent	APTES Concentrations and Incubation Time		
96% EtOH	1%, 10 min	2%, 20 min	4%, 60 min
1 mM acetic acid	1%, 10 min	2%, 20 min	4%, 60 min

## Supplementary Materials

The following are available online https://www.mdpi.com/1424-8220/20/11/3163/s1, Figure S1: Schematic illustration of possible APTES configurations after silanization, Figure S2: Surface roughness of plasma cleaned silicon oxide wafer.
