# Supplementary material for: Impact of Silanization Parameters and Antibody Immobilization Strategy on Binding Capacity of Photonic Ring Resonators
Source: Sensors (Basel). 2020 Jun 2;20(11):3163. doi: 10.3390/s20113163 (PMC7309079; doi:10.3390/s20113163)
Supplement: Supplementary file 1 [file sensors-20-03163-s001.pdf]

Supplementary information:

Impact of silanization parameters and antibody immobilization strategy on binding capacity of photonic ring resonators

Nina B. Arnfinnsdottir [1,*], Cole A. Chapman [2], Ryan C. Bailey [2], Astrid Aksnes [3] and Bjørn T. Stokke [4]

1 Department of Physics, Center for Quantum Spintronics, NTNU Norwegian University of Science and Technology, NO-7491 Trondheim, Norway; nina.arnfinnsdottir@ntnu.no
2 Department of Chemistry, University of Michigan, 930 North University Avenue, Ann Arbor, MI 48109, United States; coleac@umich.edu (C:A.C); ryancb@umich.edu (R.C.B)
3 Department of Electronic Systems, NTNU Norwegian University of Science and Technology, Trondheim NO-7491, Norway; astrid.aksnes@ntnu.no
4 Department of Physics, Division of Biophysics and Medical Technology, NTNU Norwegian University of Science and Technology, NO-7491 Trondheim, Norway; bjorn.stokke@ntnu.no

Figure S1: Schematic illustration of possible APTES configurations after silanization.

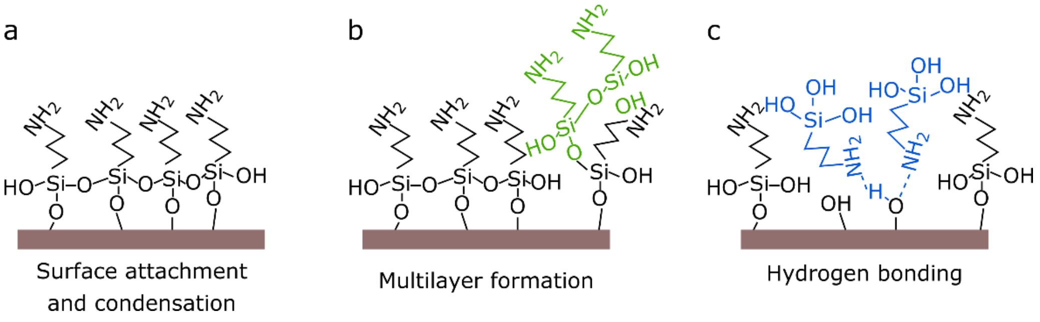

Figure S1. Schematic illustration of possible APTES configurations after silanization, (**a**); surface attachment and condensation, (**b**); multilayer formation and (**c**); hydrogen bonding.

Figure S2: Surface roughness of plasma cleaned silicon oxide wafer

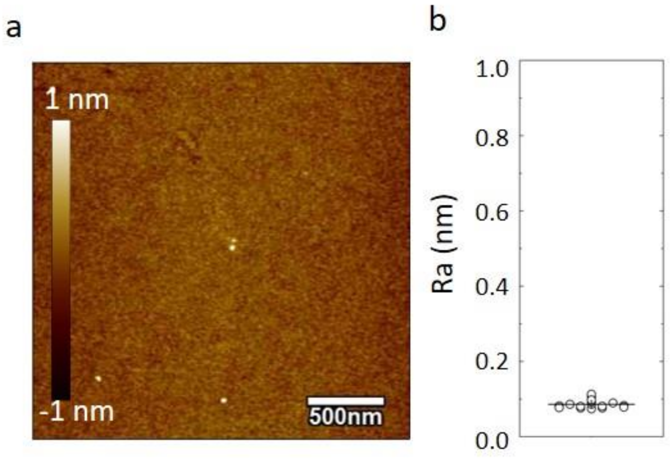

Figure S2. (**a**); AFM topograph of a plasma cleaned silicon oxide substrate. (**b**); The average surface roughness of plasma cleaned silicon oxide substrates as exemplified in (a). The average is calculated based on four separate areas imaged on each of four samples.
